# Indigenous narratives of mental illness: narratives from Naga tribes

**DOI:** 10.3389/fsoc.2025.1644475

**Published:** 2025-08-15

**Authors:** Kiniholi Yepthomi, Saranya T. S., Sandeep Kumar Gupta

**Affiliations:** ^1^School of Liberal Studies, CMR University, Bangalore, India; ^2^Department of Psychology, School of Liberal Studies, CMR University, Bangalore, India

**Keywords:** indigenous knowledge, Naga tribal healing practices, mental health and culture, community mental health, traditional healing for mental illness, traditional healing knowledge

## 1 Introduction

Native viewpoints on mental illness, particularly those based in relational, spiritual, and communal worldview have been historically denied space in mainstream psychiatric discourse. This article examines how the northeast Indian Naga people think about and treat mental distress, privileging healing as a cosmological, communal process over an individual, biomedical one. Drawing on narrative accounts and ethnographic knowledge, we seek to critically interact with indigenous epistemologies of mental health and demand a more plural, culturally sensitive framework in mental health discourse. As a team comprising a Naga scholar, a psychologist, and a researcher in indigenous knowledge systems, our perspectives are shaped by both lived experience and academic engagement. This multi-disciplinary lens informs our commitment to critically engaging with indigenous epistemologies and mental health narratives.

Psychopathology may usually be the biological picture; but for mental illness to be truly understood, it has to be culturally sanctioned and accepted. Among the Naga tribes in northeast India, mental illness is far from a problem pertaining to neurotransmitters or diagnostic nosology; it is a spirit-fueled communal experience. Our contention is that, far from being an inferior conception of mental illness, the traditional Naga healing practices constitute a culturally coherent model of emotional distress and recovery. The real worth of these healing practices rests not just on historical grounds but in the prospect that they may contribute meaningfully to present discussions on mental health (Longkumer and Rao, 2019).

Mental health among the Naga communities is deeply embedded in cosmological beliefs, clan-based responsibilities, and reciprocal social relationships. Illness is not viewed in isolation but as a disruption in the moral, spiritual, and social order. Shamanic interventions, ancestral rituals, and communal participation are central to addressing mental distress reflecting an understanding that healing is a collective process. Such practices, often dismissed in mainstream psychiatric discourse, offer alternative paradigms of care that prioritize meaning-making, social cohesion, and spiritual balance over pharmacological intervention. Ignoring these systems risks not only epistemic injustice but also the loss of context-specific insights into human suffering and resilience (Bhakuni and Abimbola, 2021).

By putting indigenous voices and stories first, this article aims to initiate discussion on the predominance of Western biomedical paradigms and the need for pluralistic mental health models. Based on narrative accounts and ethnographic impressions from Naga elders, practitioners of traditional healing, and community members, we discuss how local cosmologies interpret and treat mental distress. Instead of presenting itself as filling disciplinary lacunae, this article calls for critical examination of the necessity to decolonize psychological knowledge and incorporate culturally grounded concepts of healing into mental health discourse.

## 2 Traditional beliefs: beyond superstition

Indigenous perspectives, esp., from the vantage of modern psychiatry, are easily dismissed as superstitions. However, such an approach is far too reductive. Among the Nagas, shadowing spirit possession, soul loss, or ancestral wrath are not irrational fears; rather, they are explanations based on hundreds of years of lived experiences (Ao, 2009). Mental disturbance is viewed as something that disrupts social and spiritual harmony; it is not some flaw that is strictly internal to a person (Kumar, 2005; Singh and Indian Council of Social Science Research, 2008). The difference from Western pathology lies in the fact that the former really do emphasize interconnectedness instead of individualism.

Nagas believe in the spirit world, soul loss, or wrath of ancestors on grounds of a coherent cultural system in which mental disturbance results from a disruption in spiritual harmony (Longkumer and Rao, 2019). A household survey of 510 rural and 300 urban households in Nagaland in 2017 found that 58.9% of rural respondents and 24% of urban respondents regarded traditional healers as the major consulting group, especially in view of mood disorders thought to be caused by spirit interference (Longkumer and Rao, 2019). These systems are not merely superstitions but culturally based explanations with some diagnostic power and social relevance.

## 3 Healing as a cultural ritual, not a cure

Traditional Naga methods of healing gravitate more toward maintaining equilibrium than toward curing. Shamans and healers thus assume crucial roles, not merely as therapists but as moral and spiritual guides. The ritual processes they perform whether soul retrieval, spirit appeasement, or herbal treatments all serve to reintegrate the affected individual back into the community and cosmos (Shimray, 2002). These, in essence, are symbolic acts of healing that contemporary psychotherapy imitates through narrative reconstruction and emotional catharsis (Aldwin, 2016).

In Naga culture, mental health is community-oriented, wherein family, clan, and village heal together. Longkumer and Rao (2019) study substantiates that mental distress is addressed by the collective, with rigorous follow-ups undertaken and the entire village involved in supporting the patient. This resembles peer-supported learning approaches today: for instance, SCERT Nagaland incorporates peer counseling and movement therapy into its diploma syllabus, thus acknowledging indigenous practices in formal education settings (Longkumer and Rao, 2019).

## 4 The community's role in mental health

With its collective accountability, one of the distinctive aspects of the Naga way is that the family, clan, or village is engaged in the healing process. The traditional concept of mental illness is that it is a disturbance that affects the entire community, not just the “sick” individual. This is in stark contrast to the isolation patients may often experience in clinical systems (Adhikari, 2023). When loneliness, disconnection, and alienation are global health crises in themselves, the Naga paradigm points to the worldwide therapeutic promise held by communal care (Kienzler, 2008).

Naga traditional healing largely relies on herbal and spiritual remedies. According to Longkumer and Rao (2019), herbal remedies and manual therapies are practiced alongside psycho-spiritual techniques. For example, a mixture of honey, local roots, and substances derived from insects is used along with chanting and rituals, clearly indicating how spiritual constructs are strongly identified with ethnobotanical healing (Jamir and Watienla, 2019).

## 5 Modern psychiatry and the risk of cultural erasure

The slow decline of conventional healing practices can be traced to Western psychiatry and religious conversion, both of which have been known to stigmatize these practices as passe or pagan at times. In contemporary times, many healing rituals are simply branded as “demonic” or “pagan” (Longkumer and Rao, 2019). This is indeed a troubling line of thought. Modern psychiatry may have its merits, but it often shows little regard for native epistemologies or ways of knowing (Gone, 2013). The question, however, is not about choosing one, but rather creating an avenue for dialogue between science, tradition, shamans, and psychologists.

In Nagaland, the introduction of Western medicine and Christian religion has led to the marginalization of traditional healing methods. Ritu (2025) observes numerous rituals are now abandoned or labeled as ‘pagan,' with younger generations even less familiar with traditional knowledge systems. There is a warning from scholars that this failure to respectfully integrate these practices into formal mental health systems might lead to the erasure of centuries of local epistemologies (Longkumer and Rao, 2019; Ritu, 2025).

## 6 Conclusion: bridging the healing worlds

From our perspective, the traditional psychopathological model of the Naga tribes stands not only for an alternative way of understanding mental illness but also challenges Western dominance over psychiatric narratives. It makes us realize that mental health can no longer be considered as merely biochemical; it becomes existential and communal. It is time for us as researchers, mental health workers, or simply as fellow humans navigating distress to learn from models that embrace complexity, community, and culture. The future of mental health hinges upon our willingness to listen, whether it be to the doctor or to the traditional healer (Gone, 2013; Kumar, 2005).

Indeed, the traditional healer is still considered viable. Many rural and some urban residents in Nagaland still view traditional healers as effective sources of mental health care, particularly for spirit-related afflictions (Longkumer and Rao, 2019). Integration is being proposed, with only 40 percent of healers supporting such models if there is mutual respect and safeguard of intellectual property (Longkumer and Rao, 2019). WHO (WHO Strategy 2025–20234) has implemented policies to take forward the development of evidence based practice of traditional medicines, we really hope there will be more scientific integration of indigenous traditional healing practices to mental health care.
